# Looking for cues over time: A study on self-initiated monitoring in event-based and time-based prospective memory

**DOI:** 10.3758/s13421-025-01700-5

**Published:** 2025-03-13

**Authors:** G. Laera, F. Del Missier, S. Laloli, S. Zuber, M. Kliegel, A. Hering

**Affiliations:** 1 Cognitive Aging Lab, Faculty of Psychology and Educational Sciences, University of Geneva, 22 Chemin de Pinchat, 1227, Carouge, Geneva, Switzerland; 2 Geneva Musical Minds lab (GEMMI lab), University of Applied Sciences and Arts Western Switzerland HES-so, Geneva School of Health Sciences, Geneva, Switzerland; 3 Centre for the Interdisciplinary Study of Gerontology and Vulnerability, University of Geneva, Geneva, Switzerland; 4 LIVES, Overcoming Vulnerability: Life Course Perspectives, Swiss National Centre of Competence in Research, University of Geneva, Geneva, Switzerland; 5 Department of Life Sciences, University of Trieste, Trieste, Italy; 6 Department of Developmental Psychology, Tilburg School for Social and Behavioral Sciences, Tilburg University, Tilburg, The Netherlands

**Keywords:** Delayed intentions, Context, Monitoring, Cue focality, Predictability

## Abstract

Prospective memory (PM) is the ability to remember to perform an intended action in the future. In everyday life, people often have contextual information (e.g., the presence of cues) to support the completion of their PM tasks. The present study aimed to investigate how context (as probability of PM cue occurrence over time) and predictability affect PM. In two experiments, participants performed a laboratory PM task having the possibility to check the probability of the next PM cue occurrence whenever they wished; PM cue probability was manipulated to be temporally informative (predictable) or uninformative (unpredictable) on the actual PM cue occurrence. Both experiments showed that PM accuracy and cost on ongoing task performance increased with the presence of contextual information. Experiment 2 showed that this effect was independent of cue focality for PM accuracy but not for PM cost, for which the effect of context was particularly strong for non-focal compared to focal cues. Participants monitored the PM cue with uniform frequency over time, regardless of the context's predictability, and checked the probability of PM cue occurrence more often when the cue was non-focal compared to focal. This study showed the importance of contextual information in PM, highlighting the capacity of people to adapt the allocation of attentional resources systematically over time to optimize strategic monitoring and, in turn, PM performance.

## Introduction

Prospective memory (PM) is the ability to remember to perform an intended action at an appropriate moment in the future. Time-based prospective memory (TBPM) refers to remembering to perform an intended action at a specific time, whereas event-based prospective memory (EBPM) refers to remembering an intended action when a particular event occurs in the environment (Einstein et al., 1995; McDaniel & Einstein, 2000; Zuber et al., 2024). PM is an important cognitive function that plays a crucial role in everyday life and for functional independence, from remembering to pick up groceries when passing by the store, to completing a job application by a given deadline (Crovitz & Daniel, 1984; Haas et al., 2020; Hering et al., 2018; Laera et al., 2021). PM tasks are commonly assessed in laboratory settings: in EBPM tasks, people are asked to remember to perform a specific action in response to a particular external cue (i.e., PM cue), whereas in TBPM tasks, people are asked to remember to perform a specific action at a given time in the future (Einstein & McDaniel, 1990; Park et al., 1997). Both these tasks require people to engage in a background activity, referred to as the ongoing task (OT), while remembering to perform the intended action. Due to the additional clock-checking, TBPM tasks require more self-initiated monitoring processes than EBPM (Craik, 1986; Einstein et al., 1995) and, therefore, should rely more on executive functions and cognitive control processes (Joly-Burra et al., 2024; Zuber et al., 2024; Zuber & Kliegel, 2020).

In daily life, people can rely on contextual information from the environment about whether or not the PM cue is likely to appear soon (Bowden et al., 2017; Smith et al., 2017). Conceptually, the role of context in PM has been highlighted by the *dynamic multi-process model*, which suggests that both spontaneous (resource-free bottom-up memory recognition) and strategic (effortful top-down attentional control) processes can dynamically switch during task performance (Einstein et al., 2005; Einstein & McDaniel, 2005; McDaniel & Einstein, 2000; Shelton & Scullin, 2017). For example, suppose someone decides to return a book to the library on the way home; when they leave work and start driving home, seeing the advertisement of books would spontaneously evoke the memory of the intention to return the book. As the person approaches the library, they may engage in strategic monitoring, paying attention to the library’s location, ensuring that they have the book with them. In summary, the dynamic multi-process model assumes that, based on the contextual information available in the environment, people can exert and switch between spontaneous retrieval *and* strategic monitoring dynamically over time (Shelton & Scullin, 2017).

### Cue focality

PM tasks can be further distinguished according to cue *focality*, which impacts whether the detection of the PM cue can rely more on spontaneous retrieval versus requires more strategic monitoring (Einstein et al., 2005; Einstein & McDaniel, 2005; McDaniel & Einstein, 2000). For focal cues the OT execution involves similar cognitive processing related to the execution of the PM task (e.g., remembering to press a button whenever a specific word appears while performing a lexical decision task; Einstein & McDaniel, 1990) and cue detection can therefore be rather spontaneous. For non-focal tasks the processing of the PM cues does not overlap with the processing requirements of the OT (e.g., remembering to press a button whenever a specific syllable appears within the letter strings of the lexical decision task; Einstein et al., 2005) and cue detection therefore requires more strategic monitoring (Anderson et al., 2017; Einstein et al., 2005; McDaniel & Einstein, 2000).

### The role of context

Recently, several studies have shown that manipulating specific contextual features affects PM accuracy and cost – i.e., the increase in reaction times (RTs) for the OT when there is an additional PM task compared to the OT alone (Bowden et al., 2017; Cona et al., 2015). Studies converged in showing that, when people are told that the PM cue will occur at specific OT trial number intervals, this contextual information led to higher PM cost but also higher PM accuracy, compared to the “standard” condition in which participants did not receive any instruction about the PM cue occurrence (Peper & Ball, 2023). In terms of cognitive processes, Smith and colleagues (2017) suggested that, when contextual hints allowed participants to anticipate their relative proximity to an upcoming cue signaling the need to perform the target action, they strategically activate monitoring processes partly similar to the ones observed in TBPM tasks (Smith et al., 2017; for a review on the role of context in the preparatory attentional and memory processes theory, see Smith, 2017).

All the previous research presented above mainly focused on contextual effects in which participants were told in which context the cue would occur; such “on-off” situations showed that people can adapt their monitoring behavior accordingly (Bugg & Ball, 2017; Cona et al., 2015). These on-off contexts do not include the dynamic or changing probability of PM cues as they might occur in more naturalistic everyday environments, nor how people adapt their attentional resources to these changing conditions. In everyday life, people often only know that something will occur shortly without knowing the exact moment: it would not be adaptive to allocate attentional resources all the time, but rather more systematically, similarly to the monitoring behavior in TBPM tasks (Peper & Ball, 2023). Moreover, in the current PM paradigms, the contextual information was usually externally cued (e.g., by a trial counter; Block & Zakay, 2006; Labelle et al., 2009; Vanneste et al., 2016) and, as such, did not allow researchers to directly study self-initiated processes that are assumed to be involved in EBPM (Einstein et al., 2005; Einstein & McDaniel, 2005; McDaniel & Einstein, 2000; Shelton & Scullin, 2017).

### Self-initiated monitoring

To date, there is no direct measure for self-initiated monitoring processes in EBPM. The only direct empirical evidence of self-initiated monitoring processes is clock-checking in TBPM tasks, often referred to as *time monitoring*. Several studies have shown that time monitoring is strategic when it resembles a “J-shaped” pattern, meaning that participants initially check the clock only a few times and then rapidly increase clock checks as the target PM time approaches (Munaretto et al., 2022); many studies consistently showed that TBPM performance is increased when monitoring is deployed strategically (Jäger & Kliegel, 2008; Joly-Burra et al., 2022; McFarland & Glisky, 2009; Mioni & Stablum, 2014; Munaretto et al., 2022; Zuber et al., 2019). With the present study, we aimed to investigate whether predictable contextual information in EBPM tasks is used as the clock is used in TBPM tasks (Peper & Ball, 2023; Shelton & Scullin, 2017); if so, it is reasonable to argue that monitoring processes should be similar for EBPM and TBPM tasks (Peper & Ball, 2023). However, the lack of direct measures of self-initiated processes in EBPM has not yet allowed researchers to investigate this empirically.

### The present study

The present study aimed to assess whether contextual information and cue predictability affect self-initiated monitoring in EBPM tasks indicated by changes to PM cost and accuracy. To do so, we added a novel component to the standard EBPM task paradigm (Einstein & McDaniel, 1990). More precisely, we manipulated the contextual probability of the PM cue occurrence, allowing participants to check such a probability on the computer screen in a similar way to clock-checking in TBPM tasks. Furthermore, to investigate how the predictability of the PM cue influences performance, we manipulated the progression of the displayed probability of the PM cue: in the *predictable* condition, the presented probability of the PM cue linearly increased from 20% to 90% (with increments of 10%) in line with the actual proximity of the cue; in the *unpredictable* condition, the presented probability of the cue followed a pseudo-random order switching between increasing and decreasing probability that the cue occurs, thus making the probability progression uninformative of the approaching of the cue. However, in both conditions the displaying of the 90% probability informed of the imminent occurrence of the PM cue. Two experiments have been conducted to better understand whether and how context predictability and PM cue focality affected PM accuracy, strategic monitoring, and PM cost: Experiment 1 was carried out in the laboratory, whereas the Experiment 2 was an online replication of the first study, and additionally included the manipulation of PM cue focality.

## Experiment 1

The aim of Experiment 1 was to investigate self-initiated monitoring behavior in PM. Specifically, we directly compared for the first time EBPM and TBPM tasks to examine whether self-initiated monitoring changed in EBPM, establishing whether monitoring behavior in EBPM followed the same “J-shaped” pattern. Moreover, we compared EBPM tasks with and without information about the PM cue, aiming to understand whether having the possibility to check for the probability about the next PM cue occurrence (regardless of whether it was predictable or unpredictable) generated an intrinsic benefit on PM accuracy. We manipulated two variables: the first variable was *Block* (within-participants), which had three different task blocks: a standard TBPM task, a standard EBPM task without probability information signalling the incoming PM cue, and the experimental EBPM task with probability information (analyses on OT also comprised a fourth block called *OT baseline*, in which participants performed the OT without PM task; this served to calculate the PM cost). The second variable, manipulated between participants, was the *predictability* of the probability about the next PM cue occurrence (predictable vs. unpredictable).

Based on previous studies (Bowden et al., 2017; Bugg & Ball, 2017; Cona et al., 2015; Peper & Ball, 2023; Smith et al., 2017), we expected that PM performance would improve and PM cost would increase when the PM cue was supported by contextual information, and especially when supported by predictable compared to unpredictable information, supporting the idea that strategic allocation of attentional resources to the PM task (i.e., self-initiated monitoring) depend on the (predictable) context (Anderson et al., 2019; Shelton & Scullin, 2017; Smith, 2003, 2017). Furthermore, we predicted that self-initiated monitoring behavior would be strategic only when the displayed probability is predictable rather than unpredictable, perhaps resembling a similar “J-shaped” pattern of clock checks over time in the TBPM task. Diversely, in the unpredictable condition, participants might struggle to use the contextual information, since it does not linearly increase as in the predictable condition. Thus, we expected that the behavioral pattern of self-initiated monitoring in EBPM with unpredictable information showed a more uniform frequency of monitoring events over time, described by a flat linear function. In terms of cognitive processes, this pattern of results would indicate that attentional resources are deployed in a context-specific fashion (Cona et al., 2015; Peper & Ball, 2023).

### Method

#### Participants

Experiment 1 was statistically powered to detect small-to-medium differences in performance between experimental conditions. We computed required sample size a priori, using the software G*Power (Faul et al., 2007). The power analysis indicated that detecting an effect size of .25 at 95% power (two-tailed test, α = .05), would require a sample of at least 36 participants using an ANOVA test with four independent repeated PM measures (i.e., OT alone, EBPM standard, EBPM experimental, TBPM) and one factorial independent variable (context: predictable vs. unpredictable). 127 people (age range: 18–44 years; *M*_age_ = 23.22 years; *SD*_age_ = 4.40 years; 96 females and three non-binary) were tested.^1^ A part of the sample (*N* = 79) was recruited from the university in exchange for course credits; the remaining 48 participants were recruited using flyers and received a monetary remuneration (i.e., 10 Swiss Francs ≈ 10 USD). Thirty-three participants were excluded: 26 (20.47% of the total sample) reported having a history of neurological or major psychiatric disease within the last 5 weeks (e.g., epilepsy, depression, anxiety) or taking psychotropic drugs or other medication affecting the central nervous system; one participant (0.79% of the total sample) was older than 35 years; six participants (4.72% of the total sample) experienced technical problems in the task administration. The final sample comprised 94 individuals (age range: 18–35 years; *M*_age_ = 22.86 years; *SD*_age_ = 3.57 years; 73 females; *M*_education_ = 15.2 years; *SD*_education_ = 3.19 years).

#### Materials

**Fig. 1:**
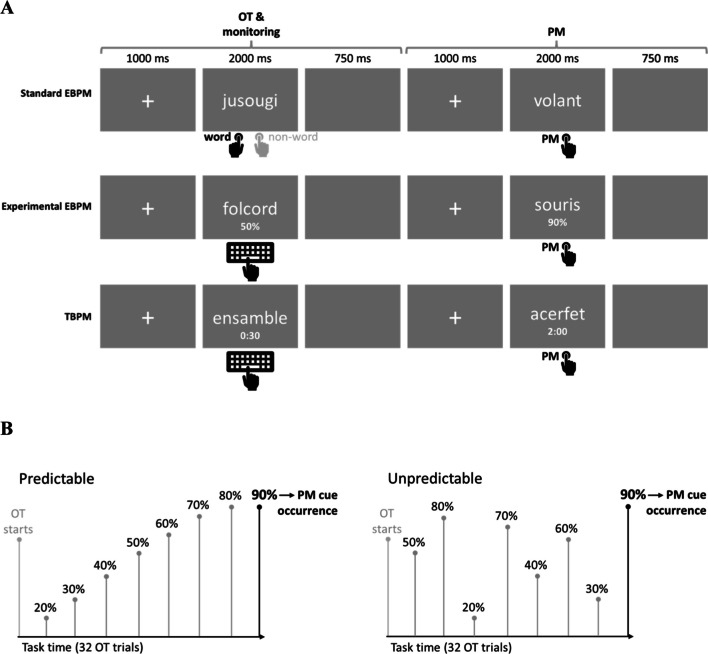
Task overview. Graphic representation of the three prospective memory tasks (standard and experimental event-based prospective memory, and time-based prospective memory, **A**). The context predictability was manipulated experimentally (**B**). In the standard event-based task (A, upper panels), participants performed a lexical decision task while remembering to press ENTER when a specific syllable appeared. In the experimental event-based task (A, middle panels), participants could check for the probability of the next cue. In the time-based task (A, lower panels), participants pressed ENTER every 2 min, with access to the clock. The probability of incoming cues was manipulated predictably (B, left panel) or unpredictably (B, right panel), with cues always occurring at “90%” probability. *EBPM* event-based prospective memory, *TBPM* time-based prospective memory, *OT* ongoing task

Participants performed three different PM tasks (TBPM, standard EBPM, and experimental EBPM) in a counterbalanced order (Mioni & Stablum, 2014); before the PM tasks, participants performed the OT baseline (i.e., the lexical decision task without PM task). All PM tasks comprised a lexical decision task as OT (Meyer & Schvaneveldt, 1971), which required participants to indicate if a string of letters presented on the screen formed a word or not. Each OT trial began with a fixation cross (1,000 ms) followed by the stimulus (2,000 ms) and a subsequent black screen (750 ms). A total of 512 stimuli (256 French words and 256 non-words) between five and eight letters long were selected, based on their highest scores in terms of standardized accuracy and lowest *z*-transformed RTs (i.e., the easiest to detect) following the rules of Ferrand (Ferrand et al., 2010). We chose to use easily detectable stimuli to ensure that the cognitive load related to the OT was low, in order to ensure that the effect of the contextual information was free from confound effects related to the difficulty of the OT task. All OT stimuli were presented in randomized order across the blocks. The total duration of each PM block was 11 min. A graphical representation of the PM tasks is shown in Fig. 1.

#### Time-based prospective memory (TBPM)

For the TBPM task block, participants had to press the ENTER key every 2 minutes while performing the OT; in total there were five TBPM target times. Participants were free to check the clock as often as they wanted by pressing the spacebar, upon which a digital clock appeared on-screen for 3 s (format: "mm:ss").

#### Standard event-based prospective memory (EBPM)

In the standard version of the EBPM task, participants had to press the ENTER button whenever they encountered the syllable “OUR” or “ANT”^2^ while performing the OT (there were five standard EBPM targets in total). In order to keep the same temporal structure between the EBPM cues and the TBPM target time, all the PM cues were presented every 2 minutes.

#### Experimental EBPM

This task was similar to the standard EBPM task except that participants could check the probability of the next PM cue to occur within the next four trials by pressing the spacebar; if they did so, the current probability (in percent) of the next PM cue occurrence appeared on-screen. Participants were randomly assigned either to the “predictable” or the “unpredictable” condition. In the “predictable” condition, the displayed probability about the next PM cue occurrence increased linearly from 20% to 90% by increments of 10%, until the PM cue occurred, and then it was reset to 20%. In the “unpredictable” condition, the displayed probability followed a pseudo-random order and did not follow a linear – predictable – increase, nor was it reset to 20% after the PM cue occurrence, as shown in Fig. 1B. However, it remained informative about the PM cue occurrence when 90% was displayed on the screen. In both the predictable and the unpredictable conditions, the probability changed every four OT trials. Participants were not informed about the condition to which they were assigned but they were aware that PM cue would follow shortly after 90%. As in the standard EBPM task, all the PM cues appeared every 2 min.

#### Procedure

All the tasks were programmed using Psychopy – version 2021.2.3 (Peirce et al., 2019); the entire procedure lasted approximately 1 hour and was administered in French. In order to control for temporal cues that could affect self-initiated monitoring, we removed clocks from the testing room, and closed the window to prevent any effect related to the day-night cycle, having only artificial lights in the room during the experiment (Barner et al., 2019; Rothen & Meier, 2017). As the participant arrived in the laboratory, the experimenter explained the aim of the study, providing an information sheet and the consent form. Once participants agreed to take part in the study, they filled out the socio-demographic questionnaire on the computer, and then performed the OT baseline after a short practice block. Then, participants were instructed to perform the three PM tasks (i.e., TBPM and standard and experimental EBPM) in a counterbalanced order; before each PM task, the practice blocks were administered once the experimenter ascertained that the participants understood the instructions. During the practice blocks, participants had to correctly perform one PM response while maintaining ≥ 80% OT accuracy, in order to begin the actual task. After the PM tasks, participants performed the Continuous performance test (AX-CPT; Braver et al., 2009), to measure attentional control (i.e., the AX-CPT is not reported in the present article because it was unrelated to the current research questions). Once people completed the AX-CPT, the follow-up questionnaire was administered, and participants were debriefed about the aims and background of the study before they left the laboratory.

### Results

Overall, we applied mixed-design ANOVAs and post hoc *t*-tests with Bonferroni’s correction for multiple testing (indicated as *p*_*adj*_). For all analyses, Greenhouse-Geisser correction was used when assumptions of sphericity were not met; moreover, we calculated the effect sizes using partial eta squared values (${\eta }_{p}^{2}$). The rejection level for inferring statistical significance was set at *p <* .05. The descriptive statistics of the dependent variables are reported in Table 1.

**Table 1:** Descriptive statistics (Experiment 1)

	Experimental conditions	OT baseline	Standard EBPM			Experimental EBPM			TBPM		Time monitoring				Probability monitoring				
		Accuracy	Accuracy	RTs (PM)	RTs (OT)	Accuracy	RTs (PM)	RTs (OT)	Accuracy	RTs (OT)	t1	t2	t3	t4	t1	t2	t3	t4	*N*
*M*	Predictable	0.68	0.85	1.027	0.801	0.89	0.996	0.821	0.99	0.727	0.82	1.26	1.72	3.52	2.13	2.1	2.34	2.37	43
	Unpredictable	0.66	0.81	1.034	0.777	0.89	1.028	0.800	0.96	0.696	0.68	1.21	1.67	3.26	1.59	1.48	1.29	1.35	46
*SD*	Predictable	0.12	0.19	0.199	0.134	0.13	0.229	0.133	0.04	0.108	0.77	0.62	0.85	1.37	1.90	1.95	2.09	2.10	
	Unpredictable	0.13	0.22	0.229	0.144	0.17	0.251	0.133	0.10	0.115	0.65	0.64	1.08	1.14	1.52	1.52	1.41	1.49	

Descriptive statistics of all outcome measures (PM accuracy and reaction times for correct PM responses, PM cost – as reaction times for correct ongoing task responses – and self-initiated monitoring) and number of participants as a function of the experimental conditions (Experiment 1); *EBPM* event-based prospective memory; *TBPM* time-based prospective memory; *RTs* reaction times (in seconds); *t1* time 1 (i.e., first 30-s interval before the PM cue or target time); *t2* time 2 (i.e., second 30-s interval before the PM cue or target time); *t3* time 3 (i.e., third 30-s interval before the PM cue or target time); *t4* time 4 (i.e., fourth and last 30-s interval before the PM cue or target time)

#### Prospective memory performance

**Fig. 2:**
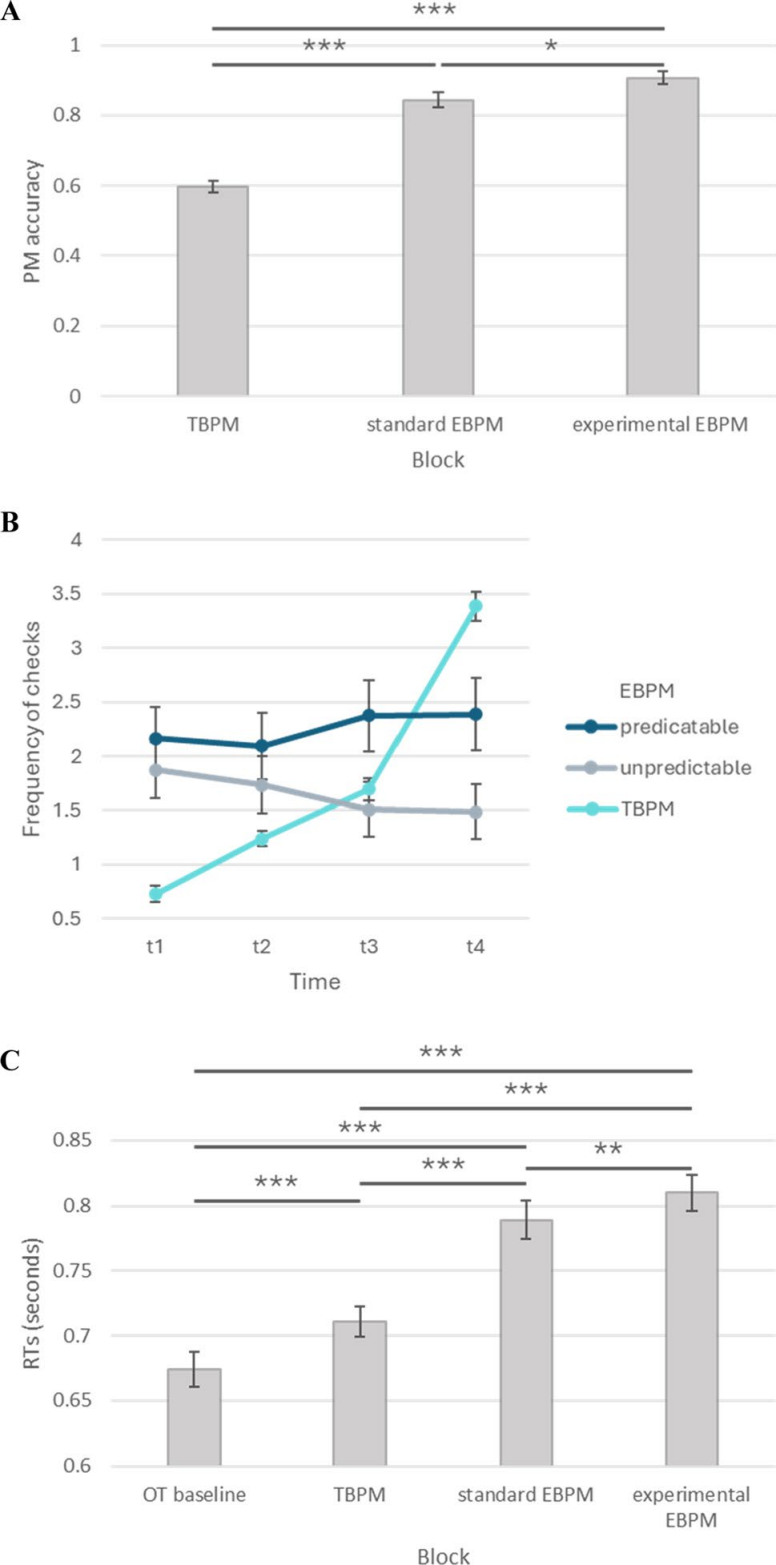
Main results (Experiment 1). *G*raphic representation of the main results from Experiment 1; specifically, results of prospective memory accuracy at the three tasks (**A**), self-initiated monitoring separately for time-based prospective memory task and experimental event-based prospective memory – both predictable and unpredictable conditions (**B**) – and prospective memory cost as reaction times for correct OT responses (**C**). Bars represent the standard error of the mean. *EBPM* event-based prospective memory; *TBPM* time-based prospective memory; *OT* ongoing task. * *p* < .05; ** *p* < .01; *** *p* < .001

In TBPM, the accuracy was computed as standardized mean proportion of the number of PM tasks accomplished within a time range of ± 3 s around the PM target time (i.e., 5% of the PM target time; Vanneste et al., 2016);^3^ in EBPM, the accuracy was computed as mean proportion of correct PM responses. The between-participants independent variable was Predictability (predictable vs. unpredictable), whereas the within-participants independent variable was Block (TBPM vs. standard EBPM vs. experimental EBPM). The analysis on PM accuracy revealed a main effect of Block, *F*(1.87, 162.81) = 81.37, *p* < .001, η^2^_*p*_ = .48, but no significant main effect of Predictability (*p* = .509) as well as no interaction of Block * Predictability (*p* = .669). Post hoc analyses revealed that participants performed worse at the TBPM task (*M* = .60, *SD* = .15) compared to both standard EBPM (*M* = .84, *SD* = .21), *t*(87) = −8.58, *p*_*adj*_ < .001, and experimental EBPM (across both predictability conditions (*M* = .91, *SD* = .16), *t*(87) = −12.95, *p*_*adj*_ < .001. Moreover, performance was significantly lower at the standard EBPM task compared to the experimental EBPM task, *t*(87) = −2.59, *p*_*adj*_ = .0034 (Fig. 2A). A separate analysis was carried out on RTs (in seconds) for correct PM responses at both EBPM tasks.^4^ We used the same between-participants independent variable Predictability (predictable vs. unpredictable), and the within-participants independent Block (standard EBPM vs. experimental EBPM) did not include TBPM. The analysis on RTs for correct PM responses at both EBPM tasks did not reveal significant effects of Block (*p* = .753) or Predictability (*p* = .391), nor any significant interaction of Block * Predictability (*p* = .794).

#### Self-initiated monitoring

Analysis on self-initiated time monitoring (measured as mean check frequency per 30-s interval with four intervals in total) was carried out using a mixed design ANOVA with Predictability (predictable vs. unpredictable) as between-participants variable, and Block (TBPM vs. experimental EBPM) and Time (t1 vs. t2 vs. t3 vs. t4) as within-participants variables. The statistical analysis showed a significant main effect of Time, *F*(2.17, 188.86) = 159.41, *p* < .001, ${\eta }_{p}^{2}$ = .65, as well as a significant Time * Predictability interaction, *F*(2.17, 188.86) = 4.32, *p* = .012, ${\eta }_{p}^{2}$ = .05, and a significant Time * Block interaction, *F*(2.25, 195.42) = 186.15, *p* < .001, ${\eta }_{p}^{2}$ = .68. Predictability (*p* = .143), Block (*p* = .292), and the Predictability * Block (*p* = .174) and Time * Block * Predictability interaction (*p* = .052) were not statistically significant. Post hoc comparisons for the interaction Time * Block revealed that participants checked the clock strategically only in TBPM tasks, as shown by the “J-shaped” growth function of time monitoring; diversely, self-initiated monitoring in EBPM followed a flat linear trend, regardless of predictability (Fig. 2B).

#### Prospective memory cost

PM cost was analyzed using RTs at correct OT trials (Anderson et al., 2019; Peper & Ball, 2023; Smith, 2003). We analyzed the data using a mixed design ANOVA. In the analysis, the between-participants independent variable was Predictability (predictable vs. unpredictable), whereas the within-participants independent variable was Block (OT baseline vs. TBPM vs. standard EBPM vs. experimental EBPM). The analysis on PM cost revealed a main effect of Block, *F*(2.31, 201.14) = 94.62, *p* < .001, ${\eta }_{p}^{2}$ = .52, but no significant main effect of Predictability (*p* = .319) as well as no Block * Predictability interaction (*p* = .934). Post hoc analyses revealed that participants were the fastest for OT baseline (*M* = .674, *SD* = .126), followed by TBPM (*M* = .711, *SD* = .112), *t*(87) = −3.97, *p*_*adj*_ < .001, then by the standard EBPM (*M* = .789, *SD* = .139), *t*(87) = −10.25, *p*_*adj*_ < .001, and finally by the experimental EBPM (*M* = .810, *SD* = .133), *t*(87) = −12.07, *p*_*adj*_ < .001. Moreover, compared to the TBPM task, participants were slower at the standard EBPM task, *t*(87) = −8.86, *p*_*adj*_ < .001, and at the experimental EBPM task, *t*(87) = −13.08, *p*_*adj*_ < .001. Finally, participants were significantly slower during the experimental EBPM than in the standard EBPM task, *t*(87) = −3.11, *p*_*adj*_ = .003 (Fig. 2C).

### Discussion

Overall, the results from Experiment 1 only partially confirmed our predictions. In summary, Experiment 1 showed that participants performed better at the EBPM task (but at a higher PM cost; Figs. 2A and C) when it was supported by contextual information, but they were not able to take advantage of cue predictability. Self-initiated monitoring (Fig. 2B) followed a “J-shaped” pattern in TBPM, in line with previous studies (Jäger & Kliegel, 2008; Joly-Burra et al., 2022; Labelle et al., 2009; Mioni & Stablum, 2014; Munaretto et al., 2022). In contrast, self-initiated monitoring followed a flat linear trend in the experimental EBPM task, with participants checking for the occurrence of the next PM cue approximately twice each 30 s. On the one hand, these results highlighted that perhaps it was not possible to predict reliably when the PM cue occurred even when the context was predictable (Munaretto et al., 2022; Shelton & Scullin, 2017); on the other hand, in presence of an unpredictable context, the uniform monitoring trend over time was in line with the hypothesis that participants found it too difficult (or not particularly useful) to track the next PM cue occurrence based on such contextual information, given the unpredictable nature of the context in this condition. In summary, Experiment 1 showed that participants performed better on the EBPM task (but at a higher PM cost) when it was supported by contextual information. To appraise the robustness of these findings, we replicated this study online including cue focality as further experimental manipulation to understand whether the uniform monitoring frequency pattern can be observed regardless of the focality of the PM cue (Anderson et al., 2017; Einstein et al., 2005; McDaniel & Einstein, 2000; Scullin et al., 2010).

## Experiment 2

The aim of Experiment 2 was to extend the results of Experiment 1 by investigating whether cue focality also affected self-initiated monitoring, PM cost, and EBPM performance, and whether the effects of context and focality were independent. Measuring the impact of focality on self-initiated monitoring can provide insights into the nature of monitoring processes affecting behavioral changes related to the context in EBPM (Scullin et al., 2013; Shelton & Scullin, 2017). Therefore, in Experiment 2, three variables were manipulated. The first variable was the *Block* (within-participants: standard EBPM vs. experimental EBPM). The second variable was the *Predictability* of the experimental PM task (predictable vs. unpredictable; between-participants). Lastly, the third variable was the PM cue *Focality* of the two EBPM tasks (focal vs. non-focal; between-participants).

We expected that participants exposed to non-focal cues might benefit more from the presence of predictable compared to unpredictable contextual information, because the predictable progression of the contextual information provides reliable information concerning the temporal distance of the PM cue and may help participants to prepare focusing on it (Ball & Bugg, 2018; Peper & Ball, 2023; Shelton & Scullin, 2017). Concerning self-initiated monitoring, Experiment 2 aimed to answer the question of whether self-initiated monitoring changed in EBPM as a function of the context and cue focality. As for Experiment 1, we expected to have a linear function, indicating a uniform distribution of the monitoring frequency over time for the cue. Given that non-focal cues are known to elicit top-down attentionally driven monitoring processes (Anderson et al., 2017; Einstein et al., 2005; McDaniel & Einstein, 2000), participants exposed to non-focal cues may report higher frequency of monitoring compared to participants exposed to focal cues, indicating that non-focal cues can indeed increase the amount of top-down self-initiated resources allocated to the PM task. This would then corroborate the assumptions of the dynamic multi-process model, indicating that such a measure of self-initiated monitoring in EBPM is related to the deployment of top-down attentional resources (Einstein & McDaniel, 2005; McDaniel & Einstein, 2000).

Finally, it is also possible that, compared to focal PM cues, monitoring for non-focal cues might benefit particularly from the presence of predictable contextual information. We expected that, when the contextual information was predictable, participants should increase the frequency of checks because predictable contexts can guide the detection of the PM cue via accumulation of progressive information in memory about the time course of the next PM cue (Peper & Ball, 2023; Smith, 2017). In contrast, unpredictable contextual information should not allow such cognitive process to take place. Diversely, focal cues should not be associated with such a benefit since they are spontaneously detected. Behaviorally, this can be observed as a specific advantage for participants exposed to the predictable context and non-focal cues, with higher PM performance and more strategic checking behavior, which may reduce PM cost compared to the unpredictable condition. The behavioral modulation induced by the interaction between cue focality and context would then support the assumptions of the dynamic multi-process model, indicating that attentional processes induced by cue focality (Einstein & McDaniel, 2005; McDaniel & Einstein, 2000) can be modulated in a context-specific manner (Shelton & Scullin, 2017).

### Methods

#### Participants

Experiment 2 was statistically powered to detect small-to-medium differences in performance between experimental conditions. We computed required sample size a priori, using the software G*Power (Faul et al., 2007). The power analysis indicated that detecting an effect size of .25 at 95% power (two-tailed test, α = .05) would require a sample of at least 72 participants using an ANOVA test with three independent repeated PM measures (i.e., OT baseline, EBPM standard, EBPM experimental) and two factorial independent variables (context: predictable vs. unpredictable; focality: focal vs. non-focal). 185 participants (age range: 18–44 years; *M*_age_ = 24.84 years; *SD*_age_ = 4.44 years; 98 females and three non-binary) were tested using Prolific (www.prolific.co). The following criteria were applied to select the sample: fluent in French, no current alcohol therapy, no head injury, no long-term health condition/disability, no chronic condition/illness, no mild cognitive impairment/dementia, no mental illness/condition, and no medication intake. Forty-three participants (~22% of the total sample) were excluded because they reported a history of neurological or major psychiatric disease within the last 5 weeks (e.g., epilepsy, depression, anxiety), or to taking psychotropic drugs or other medication affecting the central nervous system, and one participant (.54% of the total sample) because they were above 35 years old. The final sample comprised 142 individuals (age range: 18–35 years; *M*_age_ = 25.02 years; *SD*_age_ = 4.43 years; 76 females; *M*_education_ = 17.4 years; *SD*_education_ = 4.21 years). Remuneration for the participation was set at £7.51 per hour, and it was delivered according to the actual duration taken by each participant to finish the experiment. The number of participants for each experimental condition is reported in Table 2.

**Table 2:** Descriptive statistics (Experiment 2)

	Experimental conditions	Focality (EBPM)		OT alone	Standard EBPM			Experimental EBPM			Probability monitoring				
		Standard	Experimental	RTs	Accuracy	RTs (PM)	RTs (OT)	Accuracy	RTs (PM)	RTs (OT)	t1	t2	t3	t4	*N*
*M*	Predictable	Focal	Focal	0.902	0.85	0.896	0.868	0.94	0.777	0.842	1.78	1.71	2.09	2.03	16
			Non-focal	0.748	0.91	0.852	0.764	0.73	0.990	0.900	3.23	3.01	3.58	3.51	18
		Non-focal	Focal	0.809	0.62	1.121	0.862	0.92	0.819	0.824	1.72	1.65	1.64	1.75	24
			Non-focal	0.989	0.71	1.104	0.956	0.75	1.156	1.044	2.92	3.31	3.21	3.11	20
	Unpredictable	Focal	Focal	0.794	0.80	0.868	0.801	0.97	0.942	0.822	2.37	2.59	2.84	2.65	18
			Non-focal	0.829	0.88	1.022	0.801	0.79	1.217	0.915	2.52	2.45	2.51	2.33	17
		Non-focal	Focal	0.801	0.67	1.084	0.818	0.86	0.831	0.811	2.05	2.25	2.36	1.98	17
			Non-focal	0.802	0.75	1.111	0.871	0.86	1.036	0.934	3.34	3.07	2.84	2.53	13
*SD*	Predictable	Focal	Focal	0.210	0.20	0.262	0.174	0.17	0.263	0.136	2.08	2.07	2.26	2.12	
			Non-focal	0.156	0.14	0.212	0.163	0.34	0.223	0.194	2.35	2.52	2.58	2.36	
		Non-focal	Focal	0.154	0.38	0.242	0.153	0.13	0.267	0.143	1.42	1.63	1.27	1.56	
			Non-focal	0.382	0.28	0.214	0.164	0.26	0.238	0.203	2.97	3.12	3.18	2.92	
	Unpredictable	Focal	Focal	0.175	0.25	0.225	0.142	0.08	0.258	0.133	2.87	2.98	2.98	2.79	
			Non-focal	0.146	0.12	0.295	0.128	0.28	0.170	0.173	2.33	2.53	2.51	2.46	
		Non-focal	Focal	0.177	0.36	0.271	0.137	0.29	0.191	0.159	2.17	2.27	2.18	2.12	
			Non-focal	0.158	0.22	0.191	0.115	0.16	0.242	0.100	2.69	2.96	3.04	2.77	

Descriptive statistics of all outcome measures (PM accuracy and reaction times for correct PM responses, PM cost – as reaction times for correct ongoing task responses – and self-initiated monitoring) and number of participants as a function of the experimental conditions (Experiment 2). *EBPM* event-based prospective memory; *RTs* reaction times (in seconds); *t1* time 1 (i.e., first 30-s interval before the PM cue or target time); *t2* time 2 (i.e., second 30-s interval before the PM cue or target time); *t3* time 3 (i.e., third 30-s interval before the PM cue or target time); *t4* time 4 (i.e., fourth and last 30-s interval before the PM cue or target time)

#### Materials

Participants performed two different PM tasks (standard and experimental EBPM) in counterbalanced order. As in Experiment 1, all PM tasks comprised the lexical decision task as OT (Meyer & Schvaneveldt, 1971). In contrast to Experiment 1 a random blank period was introduced to avoid any temporal regularity related to the OT trials, which has been demonstrated to potentially work as a temporal cue potentially supporting time monitoring (Guo & Huang, 2019; Heathcote et al., 2015). A total of 288 stimuli (144 words and 144 non-words) between five and eight letters long were selected from the pool of stimuli used in Experiment 1, based on their highest scores in terms of standardized accuracy and lowest *z*-transformed RTs (i.e., the easiest to detect) following the rules of Ferrand (Ferrand et al., 2010). All OT stimuli were presented in fully randomized order across all the blocks. The total duration of each PM task was 9 minutes.

#### Standard EBPM

The standard EBPM task was identical to the paradigm in Experiment 1. Additionally, in the focal version, the PM cues were concrete words such as “persil” or “bureau,” whereas in the non-focal version, the PM cue was either the syllable “OUR” or the syllable “ANT”^5^ in the strings of letters while executing the OT (four EBPM responses were collected in total).

#### Experimental EBPM

This task was similar to the experimental EBPM task administered in Experiment 1, but focality was introduced as for the standard EBPM task.

#### Procedure

All the tasks were programmed using Psychopy version 2021.2.3 (Peirce et al., 2019) and hosted on Prolific via Pavlovia (https://pavlovia.org/; Bridges et al., 2020); the procedure lasted approximately 35 minutes and was administered in French. Overall, the experimental procedure was similar to Experiment 1. However, before moving onto any practice block, participants underwent an “instructional check” quiz that was not present in Experiment 1 (i.e., participants had to answer correctly to questions on the task’s instructions before proceeding; Finley & Penningroth, 2015); this quiz was helpful in ascertaining whether participants understood the instructions, since there was no experimenter in the online assessment to do so. When participants responded correctly to all the questions in the instructional check quiz, they performed the practice blocks and had to correctly perform the practice task with ≥ 80% accuracy to continue.

### Results

As in Experiment 1, we applied mixed-design ANOVAs with post hoc *t*-tests corrected using Bonferroni’s correction for multiple testing. The descriptive statistics of the dependent variables are reported in Table 2.

#### Prospective memory

**Fig. 3:**
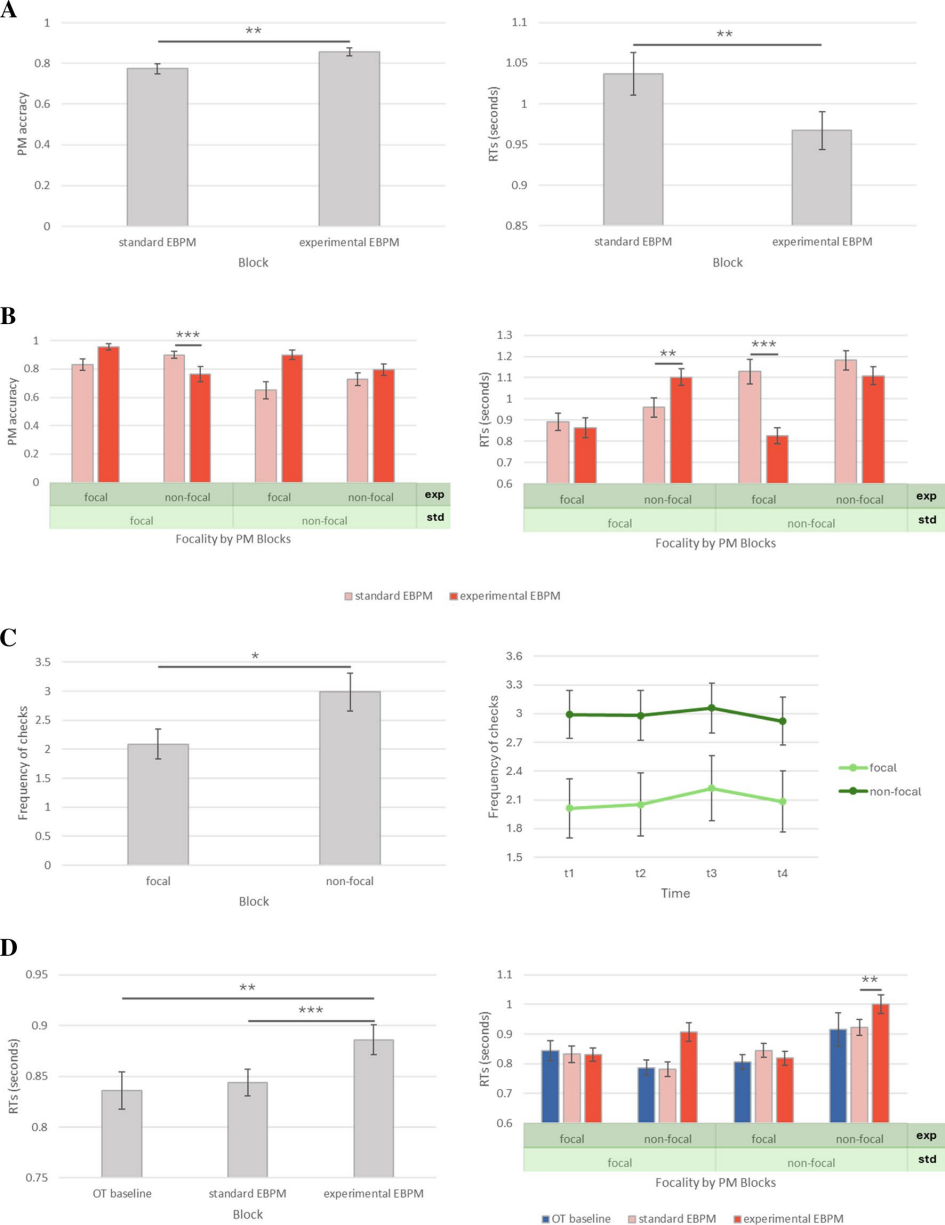
Main results (Experiment 2). *G*raphic representation of the main results from Experiment 2. Specifically, **A** represents the prospective memory performance as proportional accuracy (left panel) and as reaction times for correct responses at the PM task (right panel), as a function of the event-based prospective memory task (standard and experimental); **B** represents the prospective memory performance as proportional accuracy (left panel) and as reaction times for correct responses at the PM task (right panel) as a function of cue focality and prospective memory task; **C** represents self-initiated monitoring during experimental event-based prospective memory task as a function of focality (left panel) as well as focality and time (right panel); **D** represents reaction times for correct OT responses as a function of the event-based prospective memory task (left panel) as well as a function of task and cue focality (right panel). Bars represent the standard error of the mean. *EBPM* event-based prospective memory; *RTs* reaction times; *std* standard (EBPM); *exp* experimental (EBPM);* OT* ongoing task. * *p* < .05; ** *p* < .01; *** *p* < .001

We analyzed the data using a mixed-design ANOVA for PM performance. In the analysis, the between-participants independent variables were Predictability (predictable vs. unpredictable) and Focality (focal EBPM standard vs. non-focal EBPM standard vs. focal EBPM experimental vs. non-focal EBPM experimental), whereas the within-participants independent variable was Block (standard vs. experimental EBPM). The analysis on PM performance revealed a significant main effect of Block, *F*(1, 133) = 7.55, *p* = .007, ${\eta }_{p}^{2}$ = .05, and Focality, *F*(3, 133) = 3.07, *p* = .030, ${\eta }_{p}^{2}$ = .06, as well as a significant Block * Focality interaction, *F*(3, 133) = 8.30, *p* < .001, ${\eta }_{p}^{2}$ = .16. All other effects were not statistically significant (*p* > .05). Post hoc analyses revealed that, regardless of the Context’s condition or focality, participants performed worse at the standard EBPM (*M* = .77, *SD* = .28) compared to the experimental EBPM (*M* = .86, *SD* = .25), *t*(133) = −2.75, *p*_*adj*_ = .007 (Fig. 3A, left panel). Overall, the classic effect of focality was replicated, as participants always performed worse in the presence of non-focal (*M* = .73, *SD* = .31) compared to focal cues (*M* = .90, *SD* = .19), *t*(133) = −5.25, *p*_*adj*_ < .001 (Fig. 3B, left panel). Post hoc comparisons for the interaction Block * Focality showed that, when participants were exposed to non-focal cues during the standard EBPM task and to focal cues during the experimental EBPM task, they performed worse at the standard block (*M* = .65, *SD* = .37) compared to the experimental block (*M* = .89, *SD* = .21), *t*(133) = −4.43, *p*_*adj*_ < .001. Diversely, when participants were exposed to non-focal cues during the standard EBPM task and to focal cues during the experimental EBPM task, no difference was found (*p*_*adj*_ = .391). When cues were focal in both EBPM blocks, no difference emerged (*p*_*adj*_ = .676), as well as when cues were non-focal in both blocks (*p*_*adj*_ = 1.000).

The analysis on RTs for correct PM responses revealed a significant main effect of Block, *F*(1, 123) = 9.04, *p* = .003, ${\eta }_{p}^{2}$ = .07, and Focality, *F*(1, 123) = 15.62, *p* < .001, ${\eta }_{p}^{2}$ = .16, as well as a significant Block * Focality interaction, *F*(1, 123) = 19.74, *p* < .001, ${\eta }_{p}^{2}$ = .33. All other effects were not statistically significant (*p* > .05). Post hoc analyses revealed that, regardless of the Context’s condition or focality, participants were slower at the standard EBPM (*M* = 1.037, *SD* = .301) compared to the experimental EBPM (*M* = .967, *SD* = .276), *t*(123) = −3.01, *p*_*adj*_ = .003 (Fig. 3A, right panel). Similar to the findings of PM accuracy, the classic effect of focality was replicated, as participants were faster in the presence of focal (*M* = .884, *SD* = .257) compared to non-focal cues (*M* = 1.131, *SD* = .269), *t*(133) = −7.63, *p*_*adj*_ < .001 (Fig. 3B, right panel). Post hoc comparisons for the interaction Block * Focality showed that, when participants were exposed to focal cues during the standard EBPM task and to non-focal cues during the experimental EBPM task, they were faster at the standard block (*M* = .937, *SD* = .254) compared to the experimental block (*M* = 1.104, *SD* = .197), *t*(123) = −3.79, *p*_*adj*_ = .007. When participants were exposed to non-focal cues during the standard EBPM task and to focal cues during the experimental EBPM task, they were slower at the standard block (*M* = 1.103, *SD* = .257) compared to the experimental block (*M* = .83, *SD* = .23), *t*(123) = 7.01, *p*_*adj*_ < .001. When cues were focal in both EBPM blocks, no difference emerged, as well as when cues were non-focal in both blocks (*ps*_*adj*_ = 1.000).

#### Self-initiated monitoring

Analysis on self-initiated monitoring frequency was carried out using mixed-design ANOVAs with Predictability (predictable vs. unpredictable) and Focality (focal vs. non-focal) as between-participants variables, and Time (t1 vs. t2 vs. t3 vs. t4) as within-participants variables. The statistical analysis showed a significant main effect of Focality, *F*(1, 138) = 4.36, *p* = .039, η^2^_*p*_ = .03, as well as a significant Time * Predictability * Focality interaction, *F*(2.72, 374.94) = 2.92, *p* = .039, ${\eta }_{p}^{2}$ = .02; all other effects were not statistically significant (*p* > .05). Post hoc comparisons for the main effect of Focality revealed that participants checked the occurrence of the next PM cue less often when the PM cue was focal (*M* = 2.09, *SD* = 3.28) compared to non-focal (*M* = 2.95, *SD* = 3.46), *t*(139) = −2.17, *p*_*adj*_ < .032 (Fig. 3C, left panel); this effect was consistent in time, which indicated that participants checked the clock with a more or less constant frequency over time in both focality conditions (Fig. 3C, right panel); for this reason, post hoc comparisons for the effect of Time * Predictability * Focality interaction did not show any significant comparison (*p*_*adj*_ > .05).

#### Prospective memory cost

PM cost was inferred by analyzing RTs at correct OT trials using a mixed-design ANOVA. In the analysis, the between-participants independent variables were Predictability (predictable vs. unpredictable) and Focality (focal EBPM standard vs. non-focal EBPM standard vs. focal EBPM experimental vs. non-focal EBPM experimental), whereas the within-participants independent variable was Block (OT baseline vs. standard EBPM vs. experimental EBPM). The analysis on PM cost revealed a main effect of Block, *F*(1.50, 200) = 10.36, *p* < .001, ${\eta }_{p}^{2}$ = .07, and Focality, *F*(3, 133) = 3.71, *p* = .013, ${\eta }_{p}^{2}$ = .08, as well as a significant Block * Focality interaction, *F*(4.51, 200) = 4.88, *p* < .001, ${\eta }_{p}^{2}$ = .10; all other effects were not statistically significant (*p* > .05). Post hoc analyses revealed that, regardless of Focality and Predictability, RTs were slower during the experimental EBPM task (*M* = .886, *SD* = .174) compared to both the OT baseline (*M* = .836, *SD* = .219), *t*(133) = −3.64, *p*_*adj*_ < .001, and the standard EBPM task (*M* = .844, *SD* = .157), *t*(133) = −5.48, *p*_*adj*_ < .001 (Fig. 3D, left panel). Post hoc comparisons for the Block * Focality interaction revealed that the PM cost increased selectively when the experimental EBPM task was non-focal, regardless of the focality of the standard EBPM task: specifically, when both the standard and the experimental EBPM tasks were non-focal, PM cost was lower during the standard EBPM task (*M* = .922, *SD* = .151) compared to the experimental EBPM task (*M* = 1.001, *SD* = .177), *t*(133) = −4.41, *p*_*adj*_ = .001 (Fig. 3D, right panel); the same comparisons did not show any significant difference when both EBPM tasks were focal (*p*_*adj*_ > .050).

### Discussion

The results from Experiment 2 only partially aligned with our predictions. In line with Experiment 1 (Fig. 2A), participants performed better and were faster at the EBPM task when it was supported by probability information about cue occurrence (i.e., the context), whether predictable or not (Fig. 3A). This suggested that, for successful PM, even having some unpredictable – i.e., temporally unreliable – information about the PM occurrence is better than no information at all. Such benefit came at a significantly higher PM cost compared to the PM performance on the standard EBPM task (Fig. 3D); interestingly, standard EBPM did not seem to induce any PM cost. Focality effects were replicated (Fig. 3B) and were independent from the effect of context’s presence and its predictability in predicting PM accuracy. Self-initiated monitoring was higher for non-focal compared to focal cues and followed a linear trend indicating uniform monitoring frequency (Fig. 3C), consistent with results in Experiment 1 (Fig. 2B) and with our predictions. These results supported the dynamic multi-process model, indicating that self-initiated monitoring in EBPM is related to the deployment of top-down cognitive resources (Einstein & McDaniel, 2005; McDaniel & Einstein, 2000). Nonetheless, we also predicted that monitoring for non-focal PM cues might benefit particularly from the presence of predictable contextual information (Scullin et al., 2013; Shelton & Scullin, 2017); however, we did not observe such an advantage (i.e., higher PM performance, lower PM cost, and more strategic checking behavior). Overall, the results suggested that behavioral modulation induced by cue focality (Einstein & McDaniel, 2005; McDaniel & Einstein, 2000) was not modulated in a context-specific manner (Shelton & Scullin, 2017).

## General discussion

The main objective of the present study was to investigate the effect of contextual information and the predictability of the PM cue, and to directly compare, for the first time, behavioral measures of monitoring in EBPM and TBPM tasks. Consistently across the two experiments, results showed that PM accuracy (Figs. 2A and 3A, right panel) but also costs on OT RTs increased (Figs. 2C and 3D) with the presence of contextual information (both predictable and unpredictable) signalling the occurrence of the next PM cue; the effect of context’s predictability on PM accuracy was not significant. RTs at PM task were not consistent across experiments, because we found a significant difference between EBPM tasks in Experiment 2 but not in Experiment 1 (Fig. 3A, right panel). Self-initiated monitoring followed a linear trend in both experiments, indicating uniform monitoring frequency of the PM cue, regardless of the context’s predictability; moreover, participants checked the probability of PM cue occurrence more often when the PM cue was non-focal, compared to focal cues (Figs. 2B and 3C). Traditional effects of focality were replicated (Anderson et al., 2017; Einstein et al., 2005; McDaniel & Einstein, 2000), with participants performing better and being faster for focal compared to non-focal cues (Fig. 3B).

### Self-initiated monitoring in prospective memory

Results from Experiment 1 showed that monitoring in TBPM followed the “J-shaped” pattern, consistent with previous studies (Del Missier et al., 2021; Einstein et al., 1995; Harris & Wilkins, 1982; Mäntylä & Carelli, 2006; Munaretto et al., 2022), whereas monitoring in EBPM followed a linear trend, indicating uniform monitoring frequency. In other words, during the TBPM task participants were able to concentrate the largest amount of clock-checks temporally closer to the PM target time, whereas during the EBPM task, they distributed clock checks more or less uniformly over time, because it was not possible to reliably predict the contextual target interval (i.e. “90%”) marking the incoming PM cue occurrence, even with the presence of predictable information. This effect might be explained by the fact that clock-like progressions are very familiar to the people, hence it might have been easier to use it compared to the progressive probability of cue occurrence in EBPM, which was a new – less familiar – set of information. Results from Experiment 2 confirmed this idea, further suggesting that top-down attentional processes likely drove self-initiated monitoring and PM performance. Specifically, participants exposed to non-focal cues showed more frequent monitoring compared to participants exposed to focal cues, indicating that non-focal cues can indeed increase the amount of resources allocated to the PM task (Einstein et al., 2005; Einstein & McDaniel, 2005; McDaniel & Einstein, 2000), which, in turn, made people check the contextual probability about the next PM cue occurrence more often. This interpretation is further supported by the fact that within-participants variations in PM cost were found selectively with non-focal cues, and only during the experimental EBPM task (i.e., only when the PM cue was supported by probability information; Fig. 3D, right panel). This ultimately led to better PM performance when the task was supported by contextual information; in other words, the effect of context’s presence was related to the effect of cue focality.

In contrast to our predictions, we did not observe an advantage (i.e., higher PM performance, lower PM cost, and more strategic checking behavior) for non-focal cues in predictable contexts. Hence, this assumption of the dynamic multi-process model was not fully supported by the present data, which indicated that attentional processes induced by cue focality were not modulated in a context-specific manner (Einstein & McDaniel, 2005; McDaniel & Einstein, 2000). Nonetheless, as mentioned above, it should be noted that previous research mainly focused on contextual effects in which participants were told in which context the cue would occur. These on-off contexts did not include the changing probability of PM cues, as in the present study (Bugg & Ball, 2017; Cona et al., 2015); yet, in everyday life, people often only know that something will occur shortly but not the exact moment (Peper & Ball, 2023). Hence, the discrepancy between the present findings and the empirical framework in the literature can be explained by this different conception of the context in laboratory-based EBPM tasks. Future studies could compare static (i.e., “on-off”) contexts with dynamic changing contexts to investigate whether and how strategic monitoring changes as a function of the context dynamics.

### Contextual effects in prospective memory

The results showed that the presence of the context (as a probability of PM cue occurrence) produced significant differences in monitoring and PM performance, but such an effect was independent from focality (as the interaction Block * Predictability * Focality was not statistically significant). Although our results did not provide full support for the dynamic multi-process model, they can be explained using some of its assumptions. According to the model, focal and non-focal cues trigger bottom-up spontaneous retrieval and top-down strategic monitoring processes, respectively (Einstein et al., 2005; Einstein & McDaniel, 2005; McDaniel & Einstein, 2000). Our results suggested that both predictable and unpredictable contextual information might boost top-down strategic monitoring processes in both EBPM tasks; indeed, no matter whether the context was predictable or unpredictable, the results suggested that having just reliable information about the PM cue’s immediate occurrence (i.e., 90% of probability) is sufficient to increase performance. Behaviorally, this is evident with higher PM accuracy and a higher PM cost for non-focal EBPM tasks, but only in the presence of probability information. Moreover, higher frequency of probability checks was found for non-focal compared to focal cues. This pattern of results suggested that the presence of the context did not interact with focality for the detection of the PM cue and for retrieval of the PM task, but it affected the attentional processes involved in PM cue maintenance and retrieval independently (Kliegel et al., 2011; Peper & Ball, 2023). Hence, contextual information might act as a “facilitator” that can specifically boost top-down attentional processes mainly triggered by non-focal cues.

Another interesting point concerns whether monitoring processes are – at least partially – shared between TBPM and EBPM, as in both tasks the processing of the PM cue is independent from the processing of OT stimuli. In this sense, time-information in TBPM tasks can be considered as non-focal (i.e., outside of the attentional focus of the OT), in which the PM cue (i.e., the target time) is embodied in a predictable set of progressive metric stimuli (i.e., the clock). In the context of the present study, the clock in TBPM can be seen as contextual information like the probability of the PM cue occurrence in the experimental EBPM task (Peper & Ball, 2023). However, as mentioned above, while the clock was very familiar to the participants, allowing them to use it strategically over time, this was not possible in EBPM, in which the linear monitoring trend suggested different monitoring strategies that led to better PM performance but higher PM cost compared to the TBPM task.

## Limitations and future directions

There are some limitations to consider when interpreting our results. First, the data from the two experiments did not fully support the assumption of the dynamic multi-process model (Scullin et al., 2013; Shelton & Scullin, 2017), which requires more empirical testing. There might be other aspects relevant for PM performance, for example, motivational processes could impact performance by improving accuracy but increasing costs too (Horn & Freund, 2021; Kliegel et al., 2001, 2004); moreover, meta-cognitive aspects can also play a role in determining PM performance (Rummel et al., 2019; Schnitzspahn et al., 2011), as they can promote the engagement of more or less top-down strategic monitoring processes (Kessel et al., 2014; Wokke et al., 2016; Zuber et al., 2024).

Second, the cue in the non-focal EBPM tasks had a portion of information shared with the OT, since the PM cue was a syllable embodied in the OT stimuli; as such, PM cue monitoring and detection necessarily implied an elaboration of the OT stimuli, whereas the clock in TBPM was truly independent from the elaboration of the OT stimuli. Third, the progression of the PM cue probability was delivered in steps of 10% every four OT trials, in order to prevent participants from checking the probability at every OT trial. Perhaps more fine-grained changes in the probability of the PM cue changing every 1%, instead every 10%, could resemble a progression more similar to the clock time in TBPM.

Fourth, in this study participants were not informed about which context they would be exposed to; future studies might explicitly inform the participants about which condition they will be assigned to, because such preliminary knowledge reflects more realistically possible everyday life scenarios, as people are usually aware of whether they know (or do not know) the context in which the PM cue is embodied.

Finally, a further limitation is the nature of context across TBPM and EBPM tasks. In the TBPM tasks, the (temporal) context is always deterministic (i.e., participants were instructed that the PM cue would occur exactly after 2 minutes, and a clock was provided to exactly depict the temporal progression); in contrast, while participants in the EBPM task were informed that the cue would appear shortly after 90% completion of the ongoing task, the context was inherently more probabilistic (i.e., it is possible that participants did not perceive this cue timing as being deterministic and believed there was a smaller but still possible chance for the cue to appear at earlier points – e.g., at 20%, 30%, or 40% completion). Such perceived uncertainty could have influenced the extent to which they engaged in strategic monitoring, as suggested by previous findings (e.g., see Bugg & Ball, 2017), potentially contributing to differences observed between the TBPM and EBPM tasks (Bugg & Ball, 2017; Cona et al., 2015).

## Conclusions

This was the first study to investigate the impact of contextual information and predictability of the PM cue in self-initiated monitoring and PM; specifically, a novel task component was introduced in the EBPM task that allowed participants to check the probability of the next PM cue occurrence. Results showed that PM accuracy and PM cost increased with the presence of contextual information signalling the occurrence of the next PM cue – independently of the reliability of this information. Self-initiated monitoring frequency in EBPM tasks followed a linear trend in both experiments, indicating a uniform frequency of PM cue monitoring over time and suggesting that, in some circumstances, both predictable and unpredictable contextual information might trigger similar strategic monitoring processes.

## Data Availability

Data, metadata, codes, and materials are available in the repository of the Open Science Framework (10.17605/OSF.IO/DKPRJ); the study was registered permanently after data analysis (10.17605/OSF.IO/GXMU6).

## Footnotes

1 We collected more participants than the minimum *N* indicated by the power analysis for several reasons. One reason was to prevent losing power through possible dropouts and ensure that all participants met the exclusion criteria (which were partly evaluated post testing). Another reason was that the majority of participants were students participating for course credits; thus, according to local study regulations and ethics, all students following a specific course had the right to participate in the study. Therefore, we collected data beyond the minimal required sample size. Altogether, oversampling ensured we retained sufficient statistical power.

2 The two syllables (i.e., the non-focal PM cues) were counterbalanced between context conditions (predictable vs. unpredictable) and within EBPM blocks (standard vs. experimental), to avoid practice/facilitatory effects due to the systematic repetition of the same non-focal PM cue (Hicks et al., 2017). Moreover, we chose to use only non-focal EBPM because it has been consistently demonstrated this increases monitoring in EBPM via higher PM cost (Einstein et al., 2005); for more information about the effect of focality, see Experiment 2.

3 We choose a stringent criterion of ± 3 s because it was closer to the duration of the OT trials within the EBPM tasks (3.75 s, i.e., the maximum amount of time allowed to perform the EBPM tasks), thus making accuracy comparable between EBPM and TBPM tasks.

4 Integrating RTs analyses in addition to PM accuracy can provide further information on participants’ reactivity to the PM cue. We suggest that longer RTs on correct PM trials may indicate increased cognitive demand due to strategic monitoring for the PM task, while shorter RTs might suggest more spontaneous process retrieval with lower demand (Smith, 2003).

5 Similar to Experiment 1, the two words (i.e., the focal PM cues) and two syllables (i.e., the non-focal PM cues) were counterbalanced between PM tasks blocks (standard and experimental) as well as between context conditions (predictable vs. unpredictable), to avoid practice/facilitatory effects due to the systematic repetition of the same PM cue (Hicks et al., 2017).

## Code availability

Data were pre-processed in R – version 4.2.2 (R Core Team, 2024) – and analyses were carried out using *Jamovi* – version 2.6.23.0 (The Jamovi Project, 2024); data, metadata, codes, and materials are available in the repository of the Open Science Framework (10.17605/OSF.IO/DKPRJ).
